# Feasibility of Applying Helper-Dependent Adenoviral Vectors for Cancer Immunotherapy

**DOI:** 10.3390/biomedicines2010110

**Published:** 2014-03-10

**Authors:** Lisa M. Farzad, Masataka Suzuki

**Affiliations:** Department of Medicine, Center for Cell and Gene Therapy, Baylor College of Medicine, Houston, TX 77030, USA; E-Mail: lxfarzad@texaschildrens.org

**Keywords:** Helper-dependent adenoviral vector, immune response, pattern-recognition receptor, cancer immunotherapy

## Abstract

Adenoviruses (Ads) infect a broad range of tissue types, and derived vectors have been extensively used for gene therapy. Helper-dependent Ad vectors (HDAds), devoid of viral coding sequences, allow for insertion of large or multiple transgenes in a single vector and have been preclinically used for the study of genetic disorders. However, the clinical application of Ad vectors including HDAds for genetic disorders has been hampered by an acute toxic response. This characteristic, while disadvantageous for gene replacement therapy, could be strategically advantageous for the activation of an immune response if HDAds were used as an adjunct treatment in cancer. Cancer treatments including immunotherapy are frequently limited by the inhibitory environment produced by both tumors and their stroma, each of which express numerous inhibitory molecules. Hence, multiple inhibitory mechanisms must be overcome for development of anti-tumor immunity. The large coding capacity of HDAds can accommodate multiple immune modulating transgenes that could produce a combined effect to overcome tumor-derived inhibition and ensure intratumoral effector T-cell proliferation and function. In this review, we discuss the potential advantages of HDAds to cancer immunotherapy based on potent host immune responses to Ads.

## 1. Introduction

**Figure 1:**
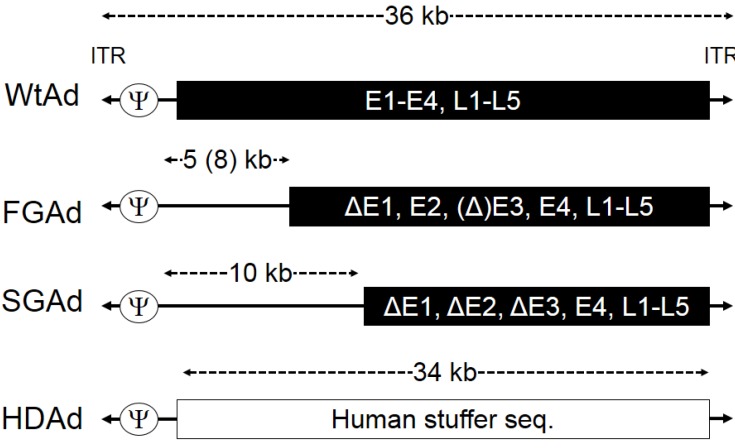
Schematic structures of adenovirus serotype 5 genome and different generations of adenoviral vectors (Ads).

Helper-dependent adenoviral vectors (HDAds) are devoid of all viral-coding sequences, and thus have the advantage of a large transgene coding capacity and, like other Ad vectors, can efficiently transduce a wide variety of cell types from various species independent of cell cycle [1]. Advances in HDAd production techniques have significantly simplified manufacture and transformed this class of agents into a realistic consideration for clinical development [2]. Since HDAds lack all viral genes in their vector DNA, cells transduced with HDAds attenuate elimination due to an adaptive cellular immune response to viral genes remaining in vector DNA, such as that seen in animal models treated with first-generation Ads (FGAds) and second-generation Ads (SGAds) [3] (Figure 1). Muruve *et al*. have shown that mice injected with FGAds induce a stronger adaptive immune response than mice injected with HDAds and immediately eliminate FGAd vector transduced cells in livers of mice [3]. This elimination is also partially dependent upon transgenes encoded in Ad vectors [3]. Attenuation of adaptive immune responses resulted the long-term expression of transgenes encoded in HDAd in transduced small and large animal models [4,5]. The HDAd cloning capacity of up to 34 kb allows for the delivery of a large therapeutic gene, multiple transgenes and/or large *cis*-acting elements to enhance, prolong and regulate transgene expression. For example, we have previously succeeded in delivering a full-length Dystrophin coding DNA, including a muscle specific promoter (total size of the expression cassette: 20 kb), using HDAd into muscles of *mdx* mice [6]. 

The HDAd vector genome remains episomal in the nuclei of transduced cells, where it associates with cellular histones and forms chromatin [7]. As a result, HDAd vectors are not associated with a risk of insertional carcinogenesis. Based on these desirable features, HDAds have been extensively studied as a therapeutic gene delivery vehicle for genetic disorders (e.g., Hemophilia A [5,8]) preclinically. However, a major obstacle to their efficacy for human gene replacement therapy is their induction of an acute inflammatory response. What represents an obstacle for gene replacement therapy could be strategically advantageous for the activation of an immune response to cancer, which is frequently limited by the inhibitory environment produced by both the tumor and its stroma, both of which express multiple inhibitory ligands, cytokines and chemokines that inhibit the intratumoral migration, proliferation and effector functions of T-cells [9]. This review will focus on how host cells sense Ad-based vectors through pattern recognition receptors (PRRs) and produce an innate immune response (that with respect to tumor therapy) could be used to mount a tumor-specific adaptive response. We will discuss how these PRR signaling pathways could contribute to cancer immunotherapy despite limiting gene therapy with Ad-based vectors. 

## 2. Production of HDAd

**Figure 2:**
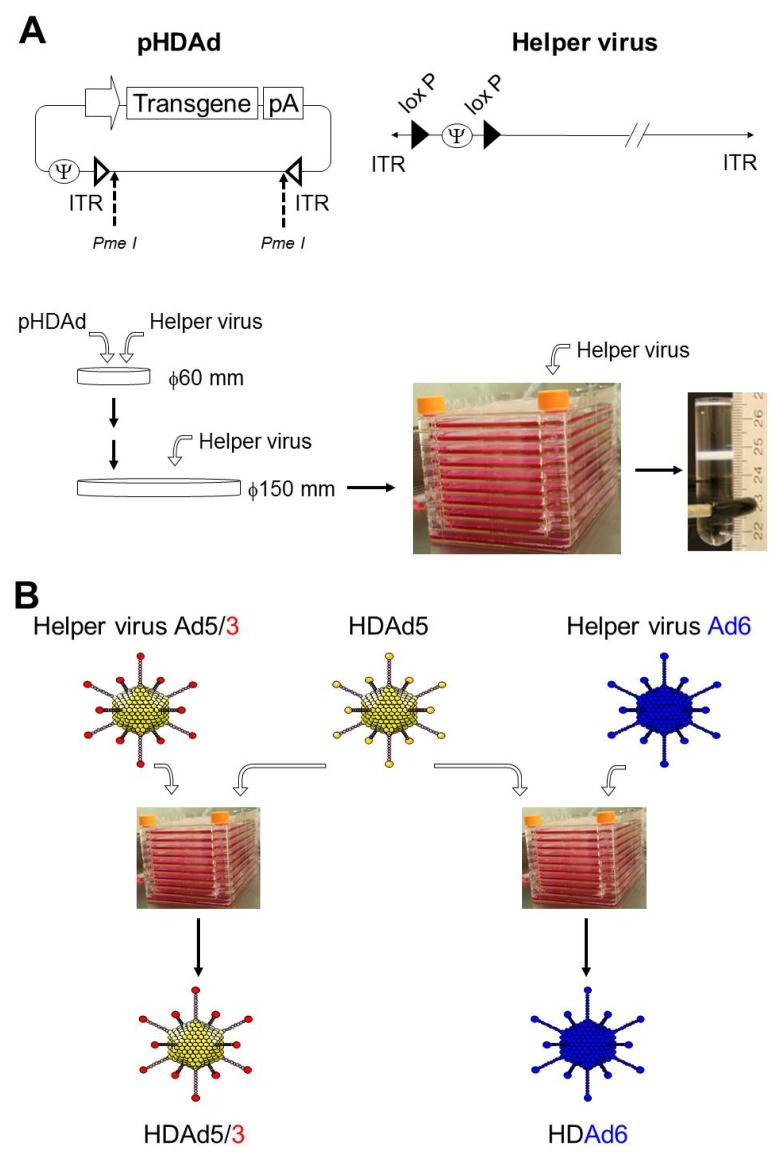
Overview of the production of Helper-dependent Ad vectors (HDAds). (**A**) A flow chart of the large-scale production of HDAd. The HDAd plasmid DNA (pHDAd) is linearized with the restriction enzyme *Pme*I before transfection to producer cell, 116 cell overexpressing Cre. HDAds are amplified by serial co-infection of helper virus and subjected to a 10-chamber cell factory. HDAd virions are purified from cell lysate by CsCl ultracentrifugation; (**B**) Generation of chimeric or different serotype HDAds. HDAd5 encoding transgene expression cassette(s) is co-infected with appropriate helper virus (chimeric or different serotype) in a 10-chamber cell factory, and produced HDAd capsid harbors helper virus dependent capsid.

Since Helper-dependent adenoviral vectors (HDAds) are devoid of all viral coding sequences and retain only the minimal viral *cis*-acting sequences necessary for vector propagation (Figure 1), co-infection of helper virus (HV) is required for rescue and large-scale production of HDAds (Figure 2A) [2,10]. The most efficient and widely used system for generating HDAd is the Cre/loxP system developed by Merck research laboratory (Patent# US5919676 A) and Graham’s group [11]. The scale up production of HDAds developed by Ng and co-workers demonstrates high infectivity and reduced HV contamination [2]. We have also recently developed an alternative system (cell factory) to obtain comparable quantities with equivalent quality to the Ng’s (spinner flask) approach, and by using adherent cells, our system reduces the technical complexity, effort, and cost compared to Ng’s approach [10]. HDAds prepared by the cell factory method exhibited low levels of helper virus contamination (0.03%–0.05%) in purified HDAds after CsCl gradient centrifugation similar to that prepared by the spinner flask method [10,12]. The viral particle-to-infectious unit ratio (vp:IU) represents the infectivity of the vector and the proportion of the total particles that are infectious. By definition, the higher vp:IU ratio corresponds to a lower infectivity per viral particle. This is a crucial parameter for a meaningful comparison between different vector preparations, because the viral particle itself mediates a dose-dependent acute toxicity, and noninfectious particles (reflected in a high vp:IU ratio) may increase risk and decrease the benefit of any treatment with Ad vectors. Therefore, the FDA has recommended that the infectivity of clinical-grade vectors for *in vivo* use have a ratio less than 30:1 of vp:IU in 293 cells [12]. We have confirmed that HDAds coding different transgenes and HDAds produced with different preparations in our method exhibit acceptable infectivity to vp ratios [10]. The dependence of HDAd particles on HV for packaging could be advantageous for differential tissue targeting, because the same HDAd backbone could be packaged by HV serotypes with different tropisms, and used to preferentially target a range of tissues types or tumors (Figure 2B). We and other groups have succeeded in producing chimeric HDAds and different serotype HDAds using appropriate HVs for targeting different tissues and cell types [6,13]. In contrast, other Ad-based vectors (e.g., FGAds, oncolytic adenoviruses (Onc.Ads)) require *de novo* generation to alter tropism. 

## 3. The Innate Inflammatory Response to Ad-Based Vectors may Contribute to Cancer Immunotherapy

**Figure 3:**
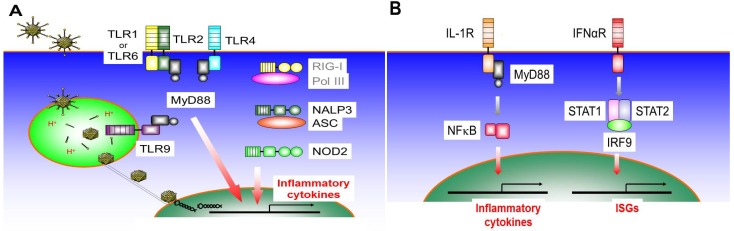
Overview of the induction of an innate immune response to Ad-based vectors. (**A**) General adenoviral infection pathway and typical pattern recognition receptors (PRRs) contribute to the induction of pro-inflammatory cytokines after infection of Ad-based vectors. After binding on the cell surface receptor (e.g., Coxsackievirus-adenovirus receptor), a clathrin-coated vehicle containing the Ad particle is formed, and clathrin-mediated endocytosis occurs. Naked capsid released from endosome interacts with microtubules and dynein motors. Capsid docks at nuclear pore and passes viral DNA (vector DNA) through nuclear pores; (**B**) Amplification of innate cytokine induction through IL-1 receptor and/or IFNα receptor. Cytokines (IL-1, type I IFN) induced through PRRs bind to their receptors and amplify the induction of multiple cytokines and other anti-viral molecules.

Systemic administration of Helper-dependent adenoviral vectors (HDAds) induces a rapid, vp-dependent host innate immune response similar to that seen with First-generation Ads (FGAds) [3]. This acute response represents a serious obstacle to clinical translation of Ad-based vectors for genetic disorders [14]. We and others have thus dissected mechanisms responsible for the induction of the innate response using *in vitro* and *in vivo* strategies as described below. Multiple pattern recognition receptors (PRRs) and their signaling pathways have been reported to contribute to the induction of an innate immune response to Ad-based vectors (Figure 3A). This could be advantageous for Ad mediated cancer gene therapy by providing a complex adjuvant for the generation of tumor antigen-specific T-cells. We discuss signaling pathways of PRRs known to associate with Ad components (e.g., the vector DNA and viral particle) and the contribution of these signaling pathways to a cancer vaccination effect below. 

### 3.1. Toll-Like Receptor 9 (TLR9)

TLR9 recognizes unmethylated 2'-deoxyribo (cytidine-phosphate guanosine) (CpG) DNA motifs that are frequently present in bacteria and viruses, but that are rare in mammals [15]. However, TLR9 can also recognize vertebrate DNA if delivered to the endosomal compartment via a transfection reagent [16]. Thus, the location of the DNA in addition to its specific sequence, modification, or the species origin of the DNA is essential for recognition by TLR9. TLR9 signaling through the myeloid differentiation primary response gene 88 (*MyD88*) leads to induction of NF-κB dependent inflammatory cytokine production [17].

After binding to its cell surface receptor (e.g., Coxsackievirus-adenovirus receptor), a clathrin-coated vehicle containing the Ad particle is formed, and clathrin-mediated endocytosis occurs [18]. Based on this natural Ad infection pathway, endosomal PRR, TLR9 was the first reported PRR to contribute to the innate immune response to Ad vectors *in vivo* [19,20]. This TLR9 dependent innate immune response to Ad vectors was observed in dendritic cells (DCs), but not macrophages *in vitro* using primary cells isolated from TLR9 deficient (TLR9^−/−^) mice [20]. Although there was no difference in an expression of a transgene in the livers of WT and TLR9^−/−^ mice at early time points (1 or 3 days post-injection) [19,20], TLR9^−/−^ mice exhibited significantly higher transgene expression compared to WT mice at a later time point [21]. This was attributed to a decreased Th1 response to both Ad particles and transgene products in TLR9^−/−^ mice, and suggested that TLR9 signaling in dendritic cells (DCs) contributes to the induction of an innate immune response and subsequent development of a Th1 adaptive immune response to Ad vectors and transgenes [21].

The TLR9 agonist (CpG ODN) has been evaluated as a vaccine adjuvant in several malignancies including renal cell carcinoma, glioblastoma, melanoma, cutaneous T-cell lymphoma and non-Hodgkin’s lymphoma [22], and CpG ODNs have been successfully used as adjuvants in cancer vaccines when added to cancer-testis antigens emulsified in Montanide ISA 51 [23]. Recently, Cerullo *et al.* reported that an Onc.Ad bearing CpG dinucleotides enhanced the development of CTLs resulting in an improved anti-tumor response when compared to Onc.Ad lacking these sequences in animal models [24]. These results suggest that the presence of an additional TLR agonist inserted into an Ad vector would enhance the development of anti-tumor immunity. These results also suggest that Ad vector components (e.g., vector DNA) provoke an immune response through PRRs (e.g., TLR9) in tumors, contrasted to that seen in WT (healthy) animals/cells, may not be sufficient for the development of an effective cancer vaccination effect resulting from the inhibitory environment produced by both the tumor and its stroma [9].

### 3.2. Toll-Like Receptor 4 (TLR4)

Lipopolysaccharide (LPS) from Gram-negative bacteria is recognized by TLR4. TLR4 is the only TLR that recruits four adaptor proteins and activates two distinct signaling pathways: “MyD88 dependent” and “TIR-domain-containing adapter-inducing interferon-β (*TRIF*) dependent” pathways [25]. Activation of both MyD88- and TRIF-dependent pathways are required to drive robust nuclear factor-kappa B (*NF-κB*) and mitogen-activated protein (*MAP*) kinase activation with a subsequent induction of inflammatory cytokines [25]. 

Type C adenoviruses, such as the widely used Ad5 derived vector, have been shown to bind to coagulation factor X (*FX*) after systemic administration [26]. FX forms a complex with Ad hexon to bind heparan sulfate-containing glycosaminoglycans (*HSPG*s) on hepatocytes and liver macrophages [26,27]. Doronin *et al.* recently demonstrated that this Ad/FX complex is sensed by TLR4 *in vivo* and induces pro-inflammatory cytokine expression through the MyD88 signaling pathway [28]. A mutation in the Ad5 hexon that disrupts its interaction with FX attenuates TLR4 mediated cytokine expression, but also impacts hepatocyte transduction. This is because the interaction of the Ad5/FX complex with HSPGs on hepatocytes is crucial for Ad5 transduction in liver (hepatocytes) [28]. 

Lipid A molecules that target the TLR4 complex are among the most commonly used vaccine adjuvants. One additional promising candidate is monophosphoryl lipid A (MPL) molecule that lacks LPS toxicity in humans. Multiple adjuvants containing MPL have been evaluated in Phase I/II vaccine studies targeting several malignancies, including melanoma and non-small-cell lung cancer, demonstrating both safety and occasional tumor regression [29]. Previously, Lapteva *et al.* reported that the anti-tumor effect of Ad-based intratumoral vaccines was abrogated in TLR4 deficient (TLR4^−/−^) mice harboring mouse breast cancer cells, supporting the critical role of TLR4 signaling in the induction of anti-tumor immunity induced by Ad vectors [30]. Their data suggest that DCs infiltrating in the tumor are crucial to the development of anti-tumor immunity in an immunocompetent syngeneic mouse model. Activation of the TLR4 signaling pathway of DCs by Ad vectors could assist in the cross-priming of an adaptive tumor antigen-specific immune response. Since TLR4 is also expressed on T-cells and potentiates T cell activation, TLR4 signaling of T cells activated by Ad vectors may also have positive effects on tumor-specific T cells [31].

### 3.3. Toll-Like Receptor 2 (TLR2)

TLR2 recognizes a wide variety of pathogen-associated molecular patterns (PAMPs) of both Gram-positive and Gram-negative bacteria and detects lipoproteins and peptidoglycans (PGN) present in both classes of bacteria, as well as lipoteichoic acid from Gram-positive bacteria. TLR2 forms heterodimers with TLR1, TLR6 or other cell surface molecules, such as Dectin-1 or CD36 to discriminate between PAMP structures [17].

We and another group have shown that TLR2 contributes to the induction of pro-inflammatory responses after systemic administration of Ad-based vectors [21,32], although it is unclear which Ad structures are recognized by this receptor. Our data suggested that the TLR2-dependent innate immune response is signaled via MyD88 in the livers of mice injected with HDAd [21]. Appledorn *et al.* showed that FGAd-dependent TLR2 signaling can activate the NF-κB signaling pathway independent of MyD88 [32], resulting in TLR2-dependent Inhibitor κB (*IκB*) degradation and Extracellular Signal-regulated Kinase (*ERK*)1/2 phosphorylation occurring within 60 min post-injection. This result suggests that TLR2 may sense the Ad particle itself or complexes of Ad with some blood factors decorating Ad particles [33] after systemic administration. Although TLR2 deficient (TLR2^−/−^) mice injected with HDAd exhibited a significantly lower induction of pro-inflammatory responses compared with wild-type (WT) mice, TLR2^−/−^ mice showed a similar induction of the Th1 and Th2 adaptive immune response to Ad particles and transgene products compared to that of WT mice [21]. In contrast, TLR2^−/−^ mice injected with FGAd showed a significantly lower IgG response to Ad particles and transgene products [32]. These results suggest that the Ad viral gene products encoded in a FGAd vector may also enhance the development of an adaptive immune response through TLR2 signaling pathway at later stages. Of note, the vector dosages differed between our group and the other study’s group perhaps accounting for the differential output of the innate and adaptive immune responses. 

A Bacillus Calmette-Guerin (BCG) strain stimulates TLR2 and TLR4 via its mycobacterial components, including its cell wall skeleton and peptidoglycans, as well as TLR9 via its bacterial DNA [29]. BCG was approved for intravesical therapy of bladder carcinoma and superficial bladder cancer *in situ* [34]. Extensive clinical investigation of BCG in patients with bladder cancer showed anti-tumor activity in the majority of them compared to intravesical doxorubicin chemotherapy, suggesting that TLR2 signaling may also contribute to the development of an anti-tumor immunity. This result also suggests that stimulation of multiple TLR signaling pathways (with a single agent) may be beneficial in effectively inducing anti-tumor immunity. Previously, Curtin *et al.* reported that TLR2 expression on DCs is essential for priming an effective immune response to cancer cells after treatment with Ad vectors in glioblastoma syngeneic animal models [35]. They demonstrated the inability of DCs to migrate to the tumor microenvironment when treated with Ad vectors expressing thymidine kinase (*TK*) or FMS-like tyrosine kinase 3 ligand (*FLt3L*) transgenes in TLR2^−/−^ mice. Additionally, DCs lacking TLR2 failed to stimulate the proliferation and activation of tumor antigen-specific T cells (especially cytotoxic T cells) after Ad treatment. Their data highlighted the importance of TLR2 involvement in innate immunity to harness an adaptive anti-tumor immune response in cancer gene therapy with Ad-based vectors [36].

### 3.4. Nod-Like Receptors (NLRs)

NLRs are characterized by a shared domain architecture that includes a nucleotide-binding domain (NBD) and a leucine rich domain. NLRs are generally highlighted for their importance in the regulation of antimicrobial responses [37]. *NOD2* recognizes distinct building blocks of peptidoglycan (PGN), a polymer consisting of glycan chains cross-linked to each other via short peptides. Activation of NOD2 leads to a conformational change that activates receptor-interacting serine-threonine kinase 2 (*RIP2*) via cellular inhibitors of apoptosis 1 and 2 (*cIAP1* and *2*), followed by subsequent ubiquitination of NF-κB essential modulator (*NEMO*), and activation of NF-κB [38]. *NLRP3* (also known as *NALP3*, *CIAS1*, *PYPAF1*) consists of a carboxyl-terminal leucine rich repeat (LRR) domain, a central NOD domain, and an amino-terminal PYD that mainly interacts with an apoptosis-associated speck-like protein containing a CARD (*ASC*). Upon activation, NLRP3 forms a multiprotein complex, termed an “inflammasome”. After sensing the relevant signal, inflammasomes are assembled between NLRs, ASC, and pro-caspase-1. Activation of pro-caspase-1 is essential for the processing of pro-IL-1β and pro-IL-18, and for secretion of their mature active forms [37]. 

Although naked capsids of Ad particles released from endosomes interact with microtubules and dynein motors in the cytoplasm and release their DNA in the nucleus through the nuclear pore (Figure 3A) [18], multiple PRRs in the cytoplasm have been reported to sense the vector DNA and induce a pro-inflammatory cytokine expression. We and another group have reported that NOD2 and NLRP3 recognize Ad vector DNA, likely via the NBD, independent of vector sequences and induce pro-inflammatory cytokines *in vivo* [39,40]. Our data showed that HDAd injected NOD2 deficient (NOD2^−/−^) mice and infected NOD2^−/−^ mouse derived embryonic fibroblasts (MEFs) show a significant reduction of inflammatory cytokine expression at both the RNA and protein levels compared to WT mice. Although NOD2 is known to direct adaptive immune responses [41], NOD2 signaling activated by HDAd infection may minimally contribute to the development of either Th1 or Th2 adaptive immune responses to Ad particles and/or transgene products [40]. The NBD of NLRs may be involved in the induction of conformational changes and the self-oligomerization that is necessary for NLR function [37]. If this oligomerization is disrupted by binding of large molecules, such as HDAd vector DNA, NLR may be unable to fully activate down-stream signaling for the development of adaptive immune responses. Since inflammasome formation (complex of NLRP3, ASC, and pro-caspase-1) is required to process the maturation of IL-1 from proIL-1, one study (contribution of NLRP3 to Ad infection) showed that NLRP3 signaling activated by Ad vector DNA requires the ASC molecule to proceed IL-1 maturation [39].

Mutation of NOD2 or RIP2 (downstream of NOD2) disrupts the balance between commensal microbes and is associated with Crohn’s disease, an inflammatory intestinal disorder increasing the risk of colitis-associated colorectal carcinoma [37,42]. There is no report demonstrating how NLR (e.g., NOD2) signaling contributes to cancer gene therapy with Ad-based vectors. That will have to be addressed in future studies.

### 3.5. RIG-I Like Receptors (RLRs)

RLRs are a family of DExD/H box RNA helicases that function as cytoplasmic sensors of PAMPs within viral RNA. The RLRs signal downstream transcriptional factor activation in order to drive type I IFN production and antiviral gene expression eliciting an intracellular immune response to control viral infection. Retinoic acid-induced gene I (*RIG-I*) and mitochondrial antiviral signaling (*MAVS*, also known as *MDA5*) proteins mediate type I IFN production in response to cytosolic double-stranded RNA (dsRNA) or single-stranded RNA (ssRNA) containing 5'-triphosphate (5'-ppp). Whereas MDA5 preferentially recognizes high-molecular-weight dsRNA fragments, RIG-I shows a preference for shorter RNA fragments and can also bind to ssRNA [43]. 

Recently, two different groups reported that cytoplasmic DNA-dependent RNA polymerase III (Pol-III) synthesizes 5'-ppp RNA from cytoplasmic DNA derived from DNA viruses (e.g., Ad, herpes simplex virus (HSV)), and this 5'-ppp RNA is sensed by RIG-I and MAVS leading to the phosphorylation of interferon regulatory transcription factor 3 (*IRF3*) and expression of type I IFN [44,45]. Contribution of RLRs to Ad infection has been characterized with an *in vitro* system, but it is still unclear how much RLR signaling pathways contribute to the induction of pro-inflammatory cytokine expression to Ad infection *in vivo*. 

Recently Qu *et al.* reported that type I IFN signaling derived from RLR signaling contributes to poly(I:C) dependent apoptosis of gastric adenocarcinoma [46]. Type I IFN signaling through CD8α^+^ DCs is crucial for development of cancer cell-specific cytotoxic T lymphocytes (CTLs) in animal models, suggesting that type I IFN induction through RLRs activated by Ads could play an important in the development of anti-tumor immunity (especially CTLs). However, there is no report demonstrating how RLR (e.g., RIG-I) signaling contributes to cancer gene therapy with Ad-based vectors. That will need to be addressed in future studies. 

### 3.6. IL-1R Signaling Pathway

IL-1α and IL-1β signal through the IL-1 receptor type I (*IL-1RI*) on various cell types (e.g., monocytes, neutrophils, macrophages, B cell and T cell), which allows for the recruitment of a second receptor subunit, IL-1R accessory protein (*IL-1RAP*). The receptor heterodimer signals through the MyD88, interleukin-1 receptor-associated kinase 4 (*IRAK4*), TNF receptor associated factor 6 (*TRAF6*) pathway resulting in activation of NF-κB and MAPK as seen in response to TLR signaling [47] (Figure 3B). 

Ad internalization into a cell is mediated by cellular integrins [18]. Several classes of integrins, such as α5β1, αvβ3 and αvβ5, bind proteins containing an RGD amino acid motif conserved in the penton protein of Ad particles. Previously, Di Paolo *et al.* demonstrated that interaction of β3 integrin (but not β1 or β5 integrins) expressed on macrophages with the RGD motif of Ad penton induces the immediate induction of IL-1α and amplifies an innate immune response to Ad through the IL-1R/MyD88 signaling pathway after systemic administration of Ads *in vivo* [48]. This activation occurs within 30 min post-injection of Ad and is independent of TLR9 and NLRP3 inflammasomes. An IL-1 dependent innate immune response was abrogated in an Ad mutant lacking the RGD motif within the viral capsid. APC-derived IL-1 directly acts on both naïve and memory T cells to enhance their expansion and survival [47]. Although our data using MyD88 deficient (MyD88^−/−^) mice indicate that this signaling pathway is crucial for development of a Th1 adaptive immune response to HDAd [21], it is still unclear how IL-1 and its signaling contribute to the activation of T cells by Ads. 

IL-1s are abundant at tumor sites (e.g., pancreatic carcinoma, lung carcinoma, cervical carcinoma), and may significantly influence the direction of the malignant process [49]. Overexpression of IL-1β within the tumor microenvironment potentiates carcinogenesis due to local inflammatory responses that possibly contribute to an increased rate of tumor development. IL-1β of host- or tumor cell-origin is also essential for the invasiveness of existing malignant cells and for promoting angiogenesis, and the elevated expression of IL-1β is a poor prognostic factor [50]. Membrane-associated IL-1α contributes to the development of antigen-specific adaptive immune responses, rather than inflammatory responses. Membrane-associated IL-1α expressed on tumor cells induces an anti-tumor cell immunity that culminates in tumor regression. Host-derived IL-1α is also crucial in cancer immunosurveillance and in shaping the immunogenicity of malignant cells during the process of tumor progression [49]. Previously, Esandi *et al.* reported that intratumoral injection of cells producing FGAd encoding an IL-1α expression cassette show growth retardation of non-small cell lung cancer in a syngeneic rat model [51]. Their data suggested that producing sufficient levels of IL-1α in tumors initiates an immunostimulatory response to cancer cells and suppresses tumor growth at both treated and distant sites. Although IL-1α and β share a receptor, they function differently in the tumor and its stroma. If the level of secreted IL-1β can be suppressed (suppression of angiogenesis) and the expression of membrane associated IL-1α increased (development of anti-tumor immunity) in parallel in tumors, the anti-tumor effect of IL-1 might be augmented. A recombinant IL-1 receptor antagonist (*IL-1Rα*) mainly acts to neutralize secreted IL-1β [52], and has been approved by the FDA for patients with rheumatoid arthritis or colorectal carcinoma [53]. Combinatorial expression of IL-1Rα with IL-1α (or other cytokines) in tumors might be interesting to evaluate in future studies.

### 3.7. IFNαR Signaling Pathway

Type I IFNs, which in humans comprise 13 *IFNα* subtypes, as well as *IFNβ*, *IFNκ*, *IFNε*, *IFNο*, *IFNτ* and *IFNδ*, all engage the ubiquitously expressed IFNα receptor (IFNαR) complex that is composed of *IFNαR1* and *IFNαR2*. Following binding by type I IFNs, signal transduction is initiated and leads to the recruitment and phosphorylation of the signal transducers and activators of transcription (*STAT*s). STAT1 and STAT2 heterodimers associate with IFN-regulatory factor 9 (*IRF9*) to form IFN-stimulated gene factor 3 (ISGF3). This complex translocates to the nucleus to induce more than 100 IFN-stimulated genes (ISGs) from IFN-stimulated response elements (ISREs) [54] (Figure 3B). 

We recently showed that type I IFN mRNA (but not type II IFN mRNA) is induced in livers of mice within 1 hr post-injection of HDAd, and that IFNα receptor dependent amplification of cytokine expression is crucial for expression of inflammatory cytokines (e.g., IL-6) after 3 hr post-injection [55]. Similar results have been shown in mice injected with FGAds [56]. This type I IFN signaling amplifies cytokine expression in the liver independently of the nature of vector DNA sequences. Interestingly, this type I IFN signaling also induced epigenetic silencing of Ad vector transgenes at the transcriptional level *in vivo*, a phenomenon also observed with another class of DNA viral vector, HSV vectors [57]. Our data suggest that type I IFN signaling dependent promyelocytic leukemia protein (PML) bodies contribute to this epigenetic modification of vector DNA [55]. Adenoviral E4 ORF3 protein inhibits this PML dependent Ad gene silencing, in transduced cells *in vitro* [58]. Although E4 ORF3 is retained in FGAds, WT mice injected with FGAd still induced transgene silencing of FGAds in their livers. This effect might be dependent on the Ad dosage for gene transfer rather than from natural infection of Ad. 

Since recombinant IFNα is used for the treatment of some malignancies, several groups have incorporated type I IFN (*IFNα2*, *IFNβ*) or type II IFN (*IFNγ*) expression cassettes into Ad vectors to provide local expression at tumor sites and to reduce the systemic toxicity associated with infusion of recombinant IFNs. Chiocca *et al.* treated patients with malignant glioma intratumorally using a FGAd encoding human IFNβ expression cassette, that induced dose dependent tumor cell apoptosis without adverse events [59]. Hence, viral vectors encoding type I IFN expression cassettes may be a double-edged sword (provoking multiple cytokine expression as a positive effect but inducing rapid transgene silencing as a negative effect). If transient type I IFN expression proves sufficient to provoke anti-tumor immunity, type I IFN gene therapy might constitute a kind of safety switch with self-inactivation due to type I IFN signaling.

## 4. Application of HDAd for Cancer Immunotherapy

Administration of Ad vectors provokes a dose-dependent acute immune response due to the activation of multiple PRR signaling pathways in primary cells, as well as in wild-type (healthy) animals (as described above) and some of these PRRs (e.g., TLRs) may also contribute to the anti-tumoral effect of Ad cancer gene therapy described above. However, this PRR activation may not be sufficient given that tumors are immune inhibitory. Since an Ad induced anti-tumor immune response is only rarely able to fully eliminate tumors, investigators have incorporated “immunomodulatory molecules”, such as pro-inflammatory cytokines, that have successfully enhanced anti-tumor immunity (development of cancer cell-specific CTLs) induced by Ad based vectors in host animals as well as patients with malignancies [60,61,62]. Intratumoral administration of oncolytic adenoviral vectors (Onc.Ad) expressing immunomodulatory genes in animal models and in humans can induce cancer cell (tumor associated antigens)-specific CTLs that can regulate uninfected tumor cells both locally and in distant metastases [62,63,64]. Although these data suggest that recruitment of the host immune system can beneficially complement the direct oncolytic activity of adenoviruses, multiple tumor immune inhibitory mechanisms must be overcome if anti-tumor immunity is to reach its full potential. 

**Figure 4:**
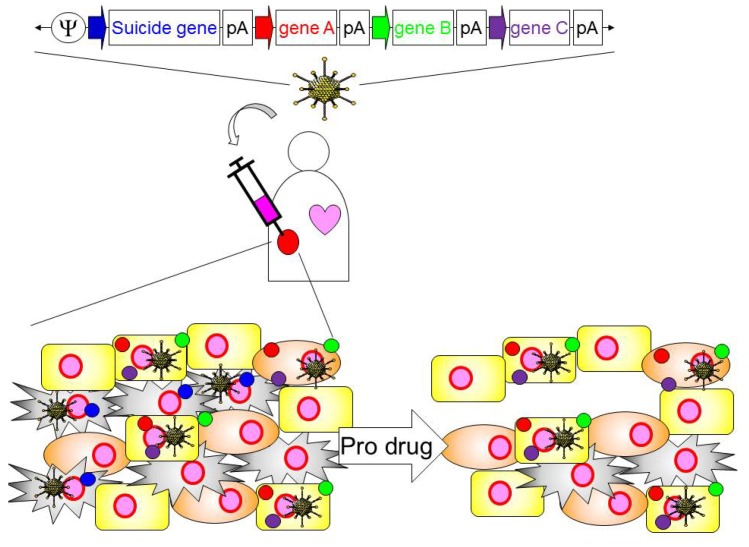
Schematic diagram of the approach of cancer gene therapy with HDAds. HDAd encoding multiple transgene expression cassettes is intratumorally injected into patient. Suicide gene is driven by cancer cell specific promoter, while other functional molecules are driven by ubiquitous and/or regulatable promoters. Patient will be treated with pro-drug, and cancer cells transduced with HDAd will result in cell death. HDAd vectors transduced in tumor stroma cells (e.g., fibroblast) still remain and express functional molecules in tumor. These functional molecules may activate the infiltrated APCs and enhance the establishment of anti-tumor immunity of host immune system.

Development of anti-tumor immunity requires the activation and regulation of multiple steps in the host immune system: (i) tumor-associated antigens (TAAs) presentation to antigen presenting cells (e.g., dendritic cells (DCs)); (ii) generation of anti-cancer effector T cells; (iii) infiltration of effector T cells into the tumor and stimulation of anti-cancer effector T cells [9]. However, both DCs and effector T cells are frequently inhibited by tumors and their stroma [65]. This suggests that optimal manipulation of potent innate and adaptive arms of the immune response for anti-tumor therapy will require the activation of proinflammatory molecules and regulation of inhibitory molecules within the tumor microenvironment. However, the expression of multiple immunomodulatory molecules in Onc.Ads compromises their titer and oncolytic functionality, because the packaging capacity of Onc.Ads is insufficient (circa 2.0 kb) and enhancing immunity may facilitate viral clearance. Thus, the increase in the immunogenicity of Onc.Ads is offset by a decline in their capacity to conditionally replicate and induce oncolysis, producing little overall net benefit [66]. This limited capacity of Onc.Ad could be overcome by combining different Onc.Ads expressing different immunomodulatory molecules. However, increasing the amount of Onc.Ad (or additional other Onc.Ads) may increase the Ad dependent side effects (toxicity). Reduction of an individual Onc.Ad amount to lower toxicity may lead to the reduction of an evoked immune response due to the immunomodulatory molecules encoded in Onc.Ads. Based on previous clinical trials with oncolytic viral vectors expressing Granulocyte Macrophage Colony-stimulating Factor (*GM-CSF*) [67,68], another obstacle of expressing multiple immunomodulatory molecules encoded within oncolytic viral vectors, including adenovirus, is that the activated immune system armed with immunomodulatory molecules may prematurely suppress therapeutic virus replication. Since HDAds by themselves lack *in vivo* tumor-lytic capacity, HDAd treatment may not promote the presentation of TAAs to antigen-presenting cells as is seen with Onc.Ad treatment. This problem could be overcome by the inclusion of a transgene with inducible lytic activity (e.g., HSV thymidine kinase, inducible caspase 9 [69]) to induce cancer cell-specific cell death, thereby resulting in the presentation of TAAs after other immunomodulatory transgenes have been evoked. It is now clear that tumors have immense heterogeneity. Intra-tumorally injected HDAds may transduce both cancer cells and stromal cells (e.g., fibroblasts). If a suicide gene, driven by a tumor-specific promoter, was encoded in an HDAd, its activation would functionally eliminate tumor cells, leaving transduced stromal cells to continue their expression of immunostimulatory molecules (Figure 4). 

**Table 1:** List of functional molecules used with Ad-based vectors in clinical trials in the USA.

Function	Gene	Cancer type	Clinical trial Code
Cytokine	*IFNβ*	Pleural Mesothelioma, Colorectal Carcinoma	NCT00299962, NCT00107861
	*IFNα2b*	Mesothelioma	NCT01212367
	*IFNγ*	B-Cell Lymphoma	NCT00394693
	*IL-12*	Breast Cancer, Colorectal Cancer, Prostate Cancer, Melanoma, Neoplasms	NCT00849459, NCT00072098, NCT00406939, NCT01397708, NCT00110526
	*IL-2*	Neuroblastoma	NCT00048386
	*MDA-7 (IL-24)*	Malignant Melanoma	NCT00116363
	*TNFα*	Esophageal Cancer, Pancreatic Cancer	NCT00051480, NCT00051467
	*GM-CSF*	Malignant Solid Tumor	NCT01598129
	*FLt3L*	Malignant Glioma	NCT01811992
Tumor suppressor	*p53*	Squamous Carcinoma, Lip and Oral Cavity Cancer, Head and Neck Carcinoma, Brain Tumors, Liver Cancer, Ovarian Cancer, Lung Cancer, Bladder Cancer, Breast Cancer	NCT00041613, NCT00064103, NCT00004041, NCT00003147, NCT00003880, NCT00003649, NCT00003167
	*REIC/Dkk-3*	Prostate cancer	NCT01197209
	*RTVP-1*	Prostatic Neoplasms	NCT00403221
Suicide molecule	*TK*	Malignant Glioma, Brain Tumors, Hepatocellular Carcinoma, Ovarian Cancer, Melanoma, Pancreatic Cancer	NCT01811992, NCT00002824, NCT00844623, NCT00638612, NCT00005057
Costimulatory molecule	*CD40L*	Malignant Melanoma, Bladder Cancer, Breast Cancer, Neoplasms, Leukemia, Lymphoma	NCT01455259, NCT00706615, NCT00504322, NCT00942409
Anti-angiogenic molecule	*Endostatin*	Head and Neck Squamous Carcinoma, Advanced solid tumors	NCT00634595, NCT00262327
Antigen	*PSA*	Prostate cancer	NCT00583752

The first decision in the design of an HDAd vector for cancer gene therapy is to identify a combination of transgenes that have complementary anti-tumor activities for incorporation into the HDAd. Table 1 summarizes transgenes that have been used with Ad-based vectors in clinical trials in the USA [70]. The majority of these trials have used a single Ad vector encoding a single transgene. However, one clinical trial for malignant glioma used 2 FGAds; one encoding an HSV TK suicide gene driven by a tumor-specific promoter and the other encoding an immunostimulatory FMS-like tyrosine kinase 3 ligand (*FLt3L*) driven by a ubiquitous promoter to augment the anti-tumor effect. Moreover, in pre-clinical glioblastoma rat models the tetracycline-dependent (TetON) switch appears to be safe (as seen by a reduction of inflammation compared to ubiquitous expression) while sustaining transgene expression (e.g., FLt3L) [71,72]. The pre-clinical and clinical findings highlighted the importance and synergy of tumor cell killing when combined with stimulation of an effective immune response [74]. Since the development of anti-tumor immunity requires the activation and regulation of multiple steps in the host immune system described above, gene(s) may need to be selected to counteract each step that is inhibited in the activation and effector phases of the immune response. First, tumor associated antigens (TAAs) released from tumor using suicide gene/pro-drug must be captured by professional antigen-presenting cells (APCs) (e.g., dendritic cells (DCs)). Combinatorial treatment of a TLR9 agonist with Onc.Ad increased tumor infiltrating DCs and correlated with therapeutic benefits in a melanoma mouse model [74], explaining why the same mouse model treated with Onc.Ad coding CpG dinucleotides enhanced TAA-specific CTLs [24]. These results suggest that stimulation of TLRs (e.g., TLR9) in tumors increases the infiltration of APCs resulting in the development of CTLs. CpG dinucleotides are short sequences and are easily incorporated into HDAd vector DNA. If the incorporation of CpG dinucleotides into HDAds is insufficient to provoke a tumor-specific adaptive immune response (e.g., CTLs), additional TLR agonist expression cassettes can be inserted. High Mobility Group Box 1 (*HMGB*1) is induced through various PRR pathways, and secreted HMGB1 binds TLR4 and TLR2 to amplify pro-inflammatory cytokine expression [75]. HMGB1 released from a dying tumor was shown to be critical for the initiation of anti-tumor immune responses in a glioblastoma animal model [35]. 

Second, tumor infiltrating APCs must be activated and matured to optimally develop TAA-specific T-cells. Granulocyte Macrophage Colony-stimulating Factor (*GM-CSF*) enhances the processing and presentation of tumor antigens by professional APCs, thus maintaining and augmenting the acquired immune response [76]. Although Onc.Ads encoding GM-CSF have produced clinical benefits in patients with various malignancies (e.g., ovarian cancer, breast cancer, lung cancer) [62], GM-CSF also promotes the immunosuppressive activity of myeloid-derived suppressor cells (MDSCs) in malignant glioma [77]. Upregulation of GM-CSF was confirmed in both human and mouse glioma microenvironments compared with normal brain or peripheral blood samples [77]. These results illustrate the point that molecules may have differential effects depending on the context. If co-expression of GM-CSF particularly promotes Myeloid-derived Suppressor Cells (MDSCs) instead of TAA presentation by APCs in certain tumor types (e.g., glioma), other molecules could be utilized activating APCs for T cell priming. CD40 ligand (*CD40*L) binds to CD40 receptors expressed on APCs and leads antigen presentation for T cell priming. Interaction of CD40L with the CD40 receptor provides costimulatory signals that trigger T lymphocyte expansion [78]. Onc.Ad expressing CD40L has been used in patients with various malignancies (e.g., colon cancer, breast cancer, prostate cancer), and some patients developed tumor-specific CTLs in blood without adverse events [79]. 

Third, tumor-infiltrating T-cells must be activated and expanded. *IL-12* is able to enhance T-helper 1 (Th1) type immunity, increase cytotoxic T-lymphocyte activity, and inhibit tumor-induced angiogenesis [80]. Treatment with IL-12 has been well studied for both immunotherapy (recombinant protein) and gene therapy using FGAds [81,82]. However, constitutive expression of IL-12, even if localized to the tumor, may cause toxicity as shown in clinical trials of recombinant IL-12 [80]. *IL-15* has proliferative effects on NK cells, effector CD8^+^, and memory phenotype CD8^+^ T cells, and a clinical trial based on application of IL-15 in tumor patients has begun [83]. 

Although while it is anticipated that combinations of “immunomodulatory molecules” affecting different steps in the activation and expansion of T-cells would enhance the development of anti-tumor immunity, the molecules necessary for effective cancer immunotherapy may require customization to individual tumor types. First, an HDAd library encoding a single functional molecule expression cassette could be made and tested to determine which combination is most effective for development of cancer cell-specific T-cells in immunocompetent animal models. After identification of the best combination for a tumor type, these transgene expression cassettes can be cloned into a single HDAd vector. 

Additional immunomodulation of the tumor microenvironment might be required to promote T-cell activation. For example, expression of immunosuppressive cytokines (e.g., *TGFβ*, *IL-10*) and/or infiltration of regulatory T-cells (Treg) in tumors have been reported and may help tumor cells evade host immune recognition [84,85]. Incorporation of inhibitors, such as soluble receptors that neutralize inhibitory cytokines [86,87], antibodies for elimination of Treg [88]) in HDAds, and co-expression of the immune modulating molecules discussed above, may augment the development of adaptive immune response.

## 5. Conclusions

Previous clinical experience with Ad vectors for malignancies has shown that adenoviral gene therapy can be effective, but increased efficacy that maintains a favorable safety profile is required. Since HDAds have been considered the safest Ad-based vector, a few groups have used them as cancer gene therapy vehicles [89,90,91]. HDAds carrying a suicide gene induced the tumor regression and long-term survival in a glioma animal model, even in the presence of systemic anti-adenovirus immunity (encountered in patients). In contrast, FGAd encoding the same expression cassette failed to elicit tumor regression in this model [92]. This result suggests that HDAd treatment may attenuate the anti-adenovirus immunity dependent elimination of vector transduced cells, resulting in long-term therapeutic gene expression compared to other Ad vectors expressing both transgene and Ad genes in transduced cells. Castro and co-workers have been evaluating the safety and efficacy of an HDAd encoding 2 functional gene expression cassettes (*TK* and *Flt3L*) in small and large animal models for clinical translation of HDAd [93].

Based on unique features of HDAds (large transgene capacity, long-term transgene expression), we are confident that HDAds will become an attractive candidate for Ad-based cancer immunotherapy. 
